# Diseases Common to Man and Animals1Read before the Haverhill Medical Club, October, 1900.

**Published:** 1901-01

**Authors:** John M. Parker

**Affiliations:** Haverhill, Mass.


### DISEASES COMMON TO MAN AND ANIMALS.[1]

By John M. Parker, D.V.S.,
HAVERHILL, MASS.

(Continued from page 736.)

*Rabies, or Hydrophobia.* This is another of the more serious of the diseases common to all animals. It seems strange that so many people should be under the impression that the disease is

[1] Read before the Haverhill Medical Club, October, 1900.

✓ OK

entirely an imaginary one and that the muzzling of dogs to prevent its spread is a farce. It is not claimed that the disease is common, or that it exists in all parts of the country, but it is more common in some districts than in others and in some seasons than in others. Because some persons have never seen a case of hydrophobia in a human being simply shows that it is not common in their part of the country, and because they have seen no such disease it does not follow that it does not exist. The positive evidence as to one outbreak is worth far more than the negative testimony of a dozen men, no matter how eminent they may be. If these persons had ever seen a characteristic case, or had ever looked into the history of an outbreak, it is hardly possible that they would still be unconvinced.

Most of you are more or less familiar with a series of cases that occurred in this State about a year and a half ago. About December 4, 1898, a dog belonging to a man named Hayes, of Ipswich, began to act in a peculiar manner, and on December 6th it ran around the neighborhood and bit several other dogs, among them one belonging to a Mr. Kinsman. The same day it was followed into the woods at Hamilton, and after a long chase was seen running around the house belonging to a Mr. Abbott, its breast and sides covered with blood and foam. It was finally shot a few hundred yards from the house.

On December 21st, or fifteen days after the attack on Mr. Kinsman's dog, when Mr. Kinsman entered his barn in the morning he saw the dog that had been bitten two weeks before by the Hayes dog biting and snarling at his cows. It had hold of one cow by the ear. Mr. Kinsman took a club and drove it off. The dog then got very ugly. It did not go out of its way to attack anyone, but it would snarl and bite if anyone got in its road.

Among others it attacked a dog belonging to one of the neighbors, a Mr. Carisle, and bit a horse on the nose. It was killed the same day. Several days later the horse began to be dull and listless and would not eat, and rather than take any chances the owner had him killed. On January 12th, or twenty-two days after the dog had been found worrying the cows in the barn, Mr. Kinsman found one of his cows acting strangely. It went into a paroxysm of rage and tried to get out of the stanchions. This had evidently been going on for some time, as there was a swelling just above its eye and at the base of the ear. It continued bellowing and striking its head; finally it was taken out from the other cows and placed in a large pen. There it smashed the door off its hinges; it bellowed and threshed its head on the floor, so that it broke off one of its horns and reduced the core to a pulp. It forced three of the boards off the side of the box-stall and took hold of a piece of board in its mouth, so that the marks of its teeth were plainly visible two days later; then it reared on its hind legs like a horse and threw itself on the floor, and finally fell down and died.

The head of this cow was sent to the laboratory and two rabbits inoculated; sixteen days after inoculation both showed marked and typical symptoms of rabies, thus confirming the earlier diagnosis.

In the meantime all dogs had been ordered muzzled for ninety days, and any dog suspected or known to have been bitten by the Kinsman dog, killed on December 21st, was ordered placed in quarantine. As a result of this order fourteen dogs were quarantined, to remain for ninety days, or until such time as the Board of Cattle Commissioners might direct.

On March 16th the dog belonging to Mr. Carisle, and bitten December 21st by the Kinsman dog—the same dog that had bitten the cow—showed evidence of rabies. This dog had been kept in a pen in the barn, and when seen by the writer showed what appeared to be rabies. He stood immovable in the middle of his pen. It was impossible to attract his attention, even by pushing a stick toward him. Occasionally he would snap in the air; at other times he would worry pieces of stick; but most of the time he would stand immovable, no one being able to attract his attention. This dog was ordered killed and the head forwarded to the laboratory. The results in this case were negative; but when we recollect the symptoms, and that this dog was bitten twelve weeks previously by the same dog that bit the cow, which was proved to be rabid by rabbit inoculation, it seems to me we must consider this a case of rabies, notwithstanding the negative results of the inoculation.

Two days later, on March 19th, word was received that a dog belonging to Mr. M. K. Abbott, of Hamilton, had died from what appeared to be rabies. The head was forwarded to the laboratory, and the rabbits inoculated developed evidence of rabies on April 18th, or twenty-five days after inoculation. On investigation it was found that Mr. Abbott lived within a few hundred yards of the place where the first dog, killed December 7th, at Hamilton, and belonging to Mr. Hayes, had been shot. It further developed that Mr. Abbott's butler had seen the Hayes dog running around the house immediately prior to its death, with blood and saliva covering its breast and sides. Of course, this does not establish absolutely the source of infection, but it does give a very probable one, as Mr. Abbott's dog was around the house at that time. It is of interest to notice that Mr. Abbott's dog died just one hundred and two days, or about three and a half months, after the Hayes dog had been killed.

At this time there were a number of dogs in quarantine. These were kept under observation until ninety days had elapsed, when, as no further evidence of rabies had appeared, they were released. No further cases have occurred in that district.

Another interesting case is reported by Dr. Cary, of Alabama, who says: "I went South eight years ago, and at that time I was rather skeptical in regard to rabies. The first case that came under my observation was that of a rabid dog biting a hog at the State Experimental Station. We had the hog put up and watched, and in about three weeks it developed a typical case of rabies by virtue of natural inoculation. That was the one thing that established the fact more in my mind than any of the ordinary reported cases. I have seen a number of cases just as well authenticated as that since that time."

The early diagnosis of a case of rabies, especially if human beings have been bitten, is an important matter. It is not altogether easy, nor, indeed, always possible, to make a diagnosis on physical examination; but, on the other hand, once the disease has developed and characteristic symptoms have shown themselves an error in diagnosis should not be made. The average period of incubation in the dog is probably from two to four weeks, although occasionally it is longer. It is seldom over three months. In the human being the period of incubation is generally much longer, the average period being probably between two and three months.

In making a post-mortem examination as an aid to diagnosis it is unfortunate that the post-mortem lesions are negative rather than positive. Even the microscopical lesions are slight. Pathologists have endeavored to point out certain changes occurring in both bloodvessels and nerve structures as pathognomonic. Several have noted various modifications in the minute structures as a result of rabies, yet it would appear that no constant and characteristic changes have been found which are absolutely characteristic of the disease. The whole matter is well summed up by Veranus A. Moore, of the B. A. I., when he points out that a histological examination is useful in a negative way, since the absence of the various lesions enumerated would strongly indicate the absence of the disease. Practically, however, to make a positive diagnosis we have got to resort to the inoculation of rabbits to get satisfactory results. The average length of time from inoculation until the first appearance of the symptoms of rabies of course varies, but in twenty-eight cases inoculated in 1898 by Dr. Frothingham, for the Massachusetts Cattle Commission, the average time was sixteen days. The shortest time was ten days and the longest thirty-six. In many cases this would be too long to wait, and where the clinical symptoms are at all characteristic it would be best not to wait for the results of inoculation before taking the Pasteur treatment.

The percentage of deaths from the bites of rabid dogs before the introduction of the Pasteur method of treatment varied greatly. Of 137 animals which were bitten by rabid dogs under observation for the last five years at the veterinary school at Berlin six only developed the disease. Zundel finds that about 25 per cent. of inoculated animals became affected, while Haubner found 40 per cent. At Alfort they have found the proportion to be about 33 per cent., and at Lyons 26 per cent.

In man Bollinger gives the proportion of human beings succumbing to bites of rabid dogs as 47 per cent.; Allbutt gives it as 16 per cent.

The probable explanation of the great variation in the number of cases that develop to the number bitten lies, at least in a great measure, in the difference in the location and severity of the bite —some being bitten more severely than others, some being bitten on the hands or face, others being bitten through fur or clothing, the teeth being wiped before penetrating the flesh, and, of course, in these cases a less amount of toxic material is introduced into the tissues. The toxic material present in the saliva also varies in amount relatively to the period of development of the disease. In some cases the toxic material is said to have been present at least three days before the symptoms of rabies have been noticed.

But whatever the cause, the average mortality does vary greatly in different localities and with different outbreaks; and whatever the mortality, it must be admitted that with the introduction of Pasteur's method of treatment the number of deaths from hydrophobia has been wonderfully reduced, the general average being under 1 per cent.

It must be remembered, however, that the Pasteur method of treatment is only a preventive measure, for if treatment is delayed too long until the symptoms have developed any treatment will be unsuccessful.

Many doubts have been cast on the Pasteur treatment, and there seems to be a general idea that it is simply a money-making scheme. It has surely stood the test of time, however. In 1896 the British House of Commons appointed a commission consisting of Sir James Paget, Dr. Lauder Brunton, Dr. George Fleming, President of the Veterinary College, London ; Sir Joseph Lister, Dr. Richard Quain, Sir Henry Roscoe, Professor Burden Sanderson, and Professor Victor Horsley to investigate the treatment for rabies as conducted by Pasteur.

" After a most searching and painstaking inquiry they reached the conclusion that Pasteur had discovered a preventive method for rabies similar to that of vaccination for variol."

The names of the men on this commission are sufficient guarantee of the reliability of its report.

So far as prevention in dogs is concerned, the muzzling of all dogs in affected areas, together with the slaughter or quarantining of exposed animals and a strict enforcement of the license law, should form the basis of all attempts to suppress any outbreak.

*Tuberculosis.* It is only since the introduction of tuberculin as a diagnostic agent that the great prevalence of tuberculosis in cattle has been fully realized. In Denmark Professor Bang reports that when tuberculosis exists in large dairies it is so prevalent that the number of cows that give a hypothermic reaction may be as high as 80 per cent. In Massachusetts the number of cows reacting to the test is very high. In 1896 there were 281 herds tested, representing 2021 dairy cows ; of this number 1098, or 54.32 per cent., were condemned and found infected. During 1897 there were 153 herds tested, including 1810 cows, and 911, or 52.54 per cent., were condemned and found infected. In these cases the herds were pretty well scattered, and probably represented fairly accurately the prevalence of the disease throughout the State.

It is interesting to notice, however, that while a large percentage of the animals in a herd may react to tuberculin, yet by far the greater nûmber of these animals are only affected to a very slight degree, and probably one of the most important facts brought to our notice through the use of tuberculin is a knowledge of the immense number of latent or undeveloped cases of tuberculosis that exist in almost every herd.

In referring to this matter Bang says : " Where tuberculosis exists it often has an extent which no one had suspected. The discovery produces at first a benumbing effect, and is always very painful to the owner of dairy stock. Because of this it cannot be made too emphatic that there is a great difference between what was formerly termed tuberculosis and what—as a result of the tuberculin test—is now indicated by the same word. When a reacting animal is butchered the butcher very often finds no trace of tuberculosis, and the veterinarian must search carefully to discover the little knots on the lymph-glands, frequently the only pathological signs present. These are especially in the retropharyngeal, mesenteric, mediastinal, and bronchial glands. The majority of reacting cows have simply latent tuberculosis. In time this may develop further, but my investigations have shown that such tuberculosis can often remain without development for years and exert no influence on the general health or functions of the animals. We cannot conclude from this observation that an animal that reacts to tuberculin is thereby condemned to advancing disease, to wasting away, and final death. The reaction simply indicates the possibility of such a result. Whether or not it will prove a reality we do not know."

On the same subject Theobald Smith says: "Tuberculin produces a temporary fever in all infected animals, whether they be in the earliest stage of the disease or the most advanced stages. In fact, the not infrequent absence of any lesion after a tuberculin reaction is explained by the statement that the focus or foci could not be detected. There are a large number of cattle slaughtered on evidence furnished by tuberculin which are in the earliest stages of the disease. A small focus in a mediastinal gland perhaps, or in one of the throat or mesenteric glands, is all that can be found.

"I believe that an infected animal is not necessarily an affected or diseased animal, and that no one can predict just what is going to become of the primary focus, whether it will gradually encroach upon neighboring organs, or become generalized, or become calcified or cicatrized."

And it is just because of this very fact, because of the large proportion of these mild cases, that it becomes so important to improve and perfect the sanitary condition of dairy barns. In this way only can these mild, incipient cases be prevented from developing.

This is, perhaps, the most important measure that can be advocated by the State looking to the prevention of bovine tuberculosis, and it should be fostered and advocated in every possible way.

In Massachusetts during the last few years the cases of advanced tuberculosis among dairy cattle have been largely weeded out. During 1895 there were 2398 cows condemned and found diseased through local inspection; of these 784, or 32.6 per cent., had generalized tuberculosis. During 1896 there were 4173 animals condemned and found diseased through the work of the local inspectors; of these 1051, or 25.1 per cent., had generalized tuberculosis. During 1897 there were 5062 animals condemned and found diseased; of these only 183, or 3.61 per cent., had generalized tuberculosis. This means, of course, that a large proportion of the bad, dangerous cases has been got rid of and the danger to the public health just so much reduced. The slight, localized cases are not necessarily dangerous. It is the cases of generalized tuberculosis, the cases showing clinical evidence of disease, either disease of the udder or otherwise, that are the most dangerous cases, and are a source of danger both to the other cattle in the barn and to human beings, and should be taken care of immediately.

This whole question is briefly and concisely reviewed by Professor Smith when he says: "It seems to me that, accepting the clinical evidence at hand, bovine tuberculosis may be transmitted to children when the body is overpowered by a large number of bacilli, as in udder tuberculosis, or when certain unknown favorable conditions exist. To prevent this from occurring a rigid periodic dairy inspection and the removal of all suspicious udder affections and all emaciated animals is as much as public authorities can at present demand. Any measures beyond these belong to agriculture, with which the public has no business to meddle without endangering the chances of gaining authority to enforce its own necessary measures. If the evidence gained by pathology in the future should reveal a greater danger than is here assumed the scientific basis of such evidence will, I think, force all additional measures needed."

This conclusion of Smith is strictly in line with the opinions expressed by the Royal Commission appointed by the British Government to investigate and report on the best methods for controlling the dangers to man from bovine tuberculosis. The report concludes: "We recommend that, for the purpose of excluding from their districts cows affected with tuberculosis of the udder or exhibiting symptoms of the disease, local authorities should be given power . . . to slaughter such cows, subject to compensation." So much is necessary for the protection of public health; the further measures necessary for the eradication of the disease from the herds are usually looked upon in Europe as purely agriculture measures. In considering the danger from the use of milk an interesting point could be raised as to the advisability of using one cow's milk as food for infants.

Statistics seem to show that tuberculosis of the udder is present in from $\frac{1}{2}$ to 1 per cent. of infected cows. Now, while it is often possible, in bad cases at least, to detect udder disease, yet many times it is utterly impossible, and if such a case did arise it would be much less dangerous if that cow's milk were to be mixed with the milk of other cows. Bollinger and others have pointed out that a dilution of 1 in 40 destroys all risk of infection from milk in the case of guinea-pigs; so that if the infected cow's milk is liberally diluted with the milk of non-infected cows the danger will practically cease to exist.

Few diseases have been more thoroughly studied than tuberculosis, yet the present state of our knowledge as to the exact relationship between human and bovine tuberculosis is rather unsatisfactory. Too much has been taken for granted, and while much work has been done in other directions the exact relationship between the two has not yet been worked out in a thoroughly scientific manner. At present it is being studied pretty thoroughly by several investigators, among them by Theobald Smith, at the Bussey Institute. Smith's idea is that in the case of infection from the bovine a study of the bacilli should show some of the characteristics of the bovine bacillus in contradistinction to a case of infection from a human being, and in summing up his report to the Massachusetts Cattle Commission on this subject he says: "We may now maintain that bovine tubercle bacilli and human bacilli as found in sputum are not identical;" and, later: "These studies will, I think, warrant one inference, however—that is, that human sputum cannot be regarded as specially dangerous to cattle, nor can it be regarded as a factor in the introduction of tuberculosis into a healthy herd of cattle."

Some time ago an interesting case came under my own notice showing the closeness of Smith's reasoning. About a year ago a dog was brought to me for treatment, showing evidence of liver and bowel trouble. After several weeks of treatment he seemed to recover, and his owner sold him for a big price. About six months later I was asked to make an autopsy, as he had died suddenly. I found generalized tuberculosis of the liver, intestines, and heart. The source of infection was seemingly through the intestinal tract, and as the dog had been bred by a man who owned a country slaughter-house I jumped to the conclusion that the dog when a puppy had been allowed to run around the slaughter-house and had eaten infected meat. Knowing Smith's interest in the matter, I forwarded the specimen to him, stating my suspicions as to the source of infection. Shortly afterward I received a letter from him stating that it appeared to him that the dog had been infected through human rather than bovine bacilli; would I not try to look carefully into the dog's early history, as he thought there must be some mistake. On looking into the matter, as requested, I made the interesting discovery that the dog had never frequented the slaughter-house, but had been trained and lived at a shanty in the woods with a number of other dogs, and that the man having charge of them had given up work in a factory and taken to this way of life *because he was a consumptive.*

In this same line, and in confirmation of Smith's theory, a Berlin investigator has made a series of studies of thirty tuberculous patients. In one patient the examination of the tubercle bacilli showed plainly the characteristics of the bovine bacillus.

These are interesting facts, and confirm the position taken by Smith that until we get positive scientific data to go upon it is impossible to be sure as to the part played by the bovine in the spread of human tuberculosis.

The *parasitic* (or second class) diseases are largely communicated to man through the ingestion of the flesh of animals, and with your permission I will reserve this subject for a later date, when I will take up the subject of slaughter-house inspection and public slaughter-houses.

(To be continued.)

